# The abundance of *Akkermansia muciniphila* and its relationship with sulphated colonic mucins in health and ulcerative colitis

**DOI:** 10.1038/s41598-019-51878-3

**Published:** 2019-10-30

**Authors:** Helen Earley, Grainne Lennon, Áine Balfe, J. Calvin Coffey, Desmond C. Winter, P. Ronan O’Connell

**Affiliations:** 1 School of Medicine, University College Dublin, Belfield, Dublin 4, Ireland; 2 Centre for Colorectal Disease, St Vincent’s University Hospital, Dublin 4, Ireland; 3 Graduate Entry Medical School, Limerick, Ireland; 4 School of Biological and Health Sciences, Technological University Dublin City Campus, Dublin, Ireland

**Keywords:** Ulcerative colitis, Dysbiosis

## Abstract

*Akkermansia muciniphila* utilises colonic mucin as its substrate. Abundance is reduced in ulcerative colitis (UC), as is the relative proportion of sulphated mucin in the mucus gel layer (MGL). It is unknown if these phenomena are related, however reduced sulphated mucins could contribute to reduced abundance, owing to a lack of substrate. The aim of this study was to quantify *A. muciniphila* within the MGL and to relate these findings with markers of inflammation and the relative proportion of sulphomucin present. Colonic biopsies and mucus brushings were obtained from 20 patients with active UC (AC), 14 with quiescent UC (QUC) and 20 healthy controls (HC). *A. muciniphila* abundance was determined by RT-PCR. High iron diamine alcian-blue staining was performed for histological analysis. Patients with AC had reduced abundance of *A. muciniphila* compared to HC and QUC. A positive association was found between *A. muciniphila* abundance and higher percentage of sulphated mucin (ρ 0.546, p = 0.000). Lower abundances of *A. muciniphila* correlated with higher inflammatory scores (ρ = 0.294 (p = 0.001)). This study confirms an inverse relationship between *A. muciniphila* and inflammation and a positive association between *A. muciniphila* abundance and percentage of sulfated mucin in the MGL.

## Introduction

The *Akkermansia* genus is present abundantly in the human gastrointestinal tract where it is believed to be a key symbiont member of the microbiota^1–5^. Since its discovery, evidence is accumulating suggesting a beneficial role for *A. muciniphila*^4,6–9^. The species has the ability to modulate host immune responses and may play a role in immune-tolerance to commensal microbes^6^.

Significant reductions in *A. muciniphila* have been demonstrated in both faecal samples and mucosal biopsies of patients with UC^8,10–12^. Germ free mice colonised with *A. muciniphila* do not develop microscopically visible inflammation, strengthening the argument for a protective role of this microbe in the setting of UC^6^. To date, the potential factors contributing to the reduced abundance of this species in UC have not been explored.

The mucus gel layer (MGL), comprised predominantly of mucins, represents the host microbial interface in the human colon. It is composed of a loosely adherent outer layer, which is home to the resident microbiota and a densely adherent inner layer which prevent bacterial penetration to the epithelium^13^. Quantitative and qualitative changes occur within the MGL in UC, including depletion of the layer, altered glycosylation and alterations of the proportions of sulphated and sialyated mucin^14–16^. Such changes alter the microenvironment in which the commensal microbiota resides, which may have implications for their survival. *A. muciniphila* utilises mucin as its substrate and the species has the propensity to produce several mucolytic enzymes, one of which is a sulfatase, which cleaves the terminal sulfate moiety of mucin^17^. Biochemical alterations in mucin such as in UC may therefore impact bacterial growth.

To date, published data pertaining to *A. muciniphila* abundance have derived from studies employing either faecal samples or whole mucosal biopsies. A degree of spatial variation exists across the cross sectional axis of the human colon, with distinct microbial communities residing in the luminal contents, mucus and mucosa^18–20^. Therefore, neither of these sampling methods reflect the microbial composition at the host microbial interface. Brushings of the MGL facilitate optimum study of the innate microbiota that is stable over space and time, owing to the fact that the layer is enriched with microbial DNA and communities within it are less susceptible to changes related to dietary factors when compared with other sampling methods^20,21^.

The aim of this study was to perform quantitative analysis of *A. muciniphila* within the MGL in health and in patients with UC and to correlate these findings with markers of colonic inflammation and the relative proportion of sulphated mucin present within the MGL.

## Materials and Methods

### Ethical approval, patient recruitment and sample collection

Ethical approval was obtained from the St. Vincent’s University Hospital Ethics and Medical Research Committee Version 7, 2012. All methods were carried out in accordance with this. All participants were over 18 years of age and gave written informed consent. Three patient cohorts were established for this study: healthy controls (HC), patients with quiescent UC (QUC) and patients with active UC (AC).

Healthy volunteers were recruited before undergoing routine diagnostic day case colonoscopy. No macroscopic evidence of mucosal pathology was evident in these individuals. Patients were excluded from the study if they had a history of antibiotic usage or hospital admission in the six weeks prior to colonoscopy, personal history of irritable bowel syndrome (IBS), indeterminate colitis, gastrointestinal malignancy or previous colorectal surgery.

Patients with quiescent UC were identified as having been previously diagnosed with histologically confirmed UC and who were undergoing surveillance colonoscopy. Exclusion criteria were as above or evidence of UC associated dysplasia. The bowel preparations received by all patients undergoing colonoscopy were polyethylene glycol and sodium picosulphate based.

Patients in the AC cohort were recruited prior to undergoing surgical resection for disease refractory to medical management or those with AC failing to respond to rescue therapy (intravenous steroids, biologics or cyclosporine). Patients had not received bowel preparation prior to undergoing surgery, but had received a single dose of intravenous antibiotics prior to induction of anaesthesia, as per Hospital protocol. MAYO scores of disease severity^22^ were available for all patients included in the study. These were calculated as outlined in Supplementary Table 1.

Patients were excluded from further consideration if they had been prescribed oral or intravenous antibiotics in the previous 3 months, had a history of colon cancer, colonic resection or active GI bleeding, were residents of a long-term care facility or had been a hospital inpatient within the previous 6 weeks.

To obtain mucus brushings, a Hobbs’ Microbiological Protected Specimen Brush (PSB) (Hobbs Medical Inc., CT 06076, U.S.A,) was advanced at colonoscopy in a protective sheath, deployed under direct vision and brushed multiple times until coated, as previously described^20^. It was closed under direct vision and retracted through the colonoscope port. Radial Jaw® 3 biopsy forceps (Boston Scientific, Cork, Ireland) were used to target a region immediately adjacent to that which had been directly sampled by the PSB and a whole mucosal biopsy retrieved for histological analysis. Samples were stored in sterile 1.5 ml micro-centrifuge tubes on dry ice until DNA extraction.

DNA extracts were isolated from colonic mucus brushings and stored in sterile micro-centrifuge tubes at −20 °C. DNA was extracted using a Qiagen DNA mini kit (Qiagen, Hilden, Germany). Paired formalin-fixed and paraffin-embedded mucosal biopsies were also obtained for histological analysis. The biobank of samples consisted of DNA from 20 HCs, 14 patients with QUC and 20 patients with AC. For each patient, samples were collected from four areas of the colon; caecum, transverse colon, left colon and rectum.

### Histological analysis of specimens

**Figure 1:**
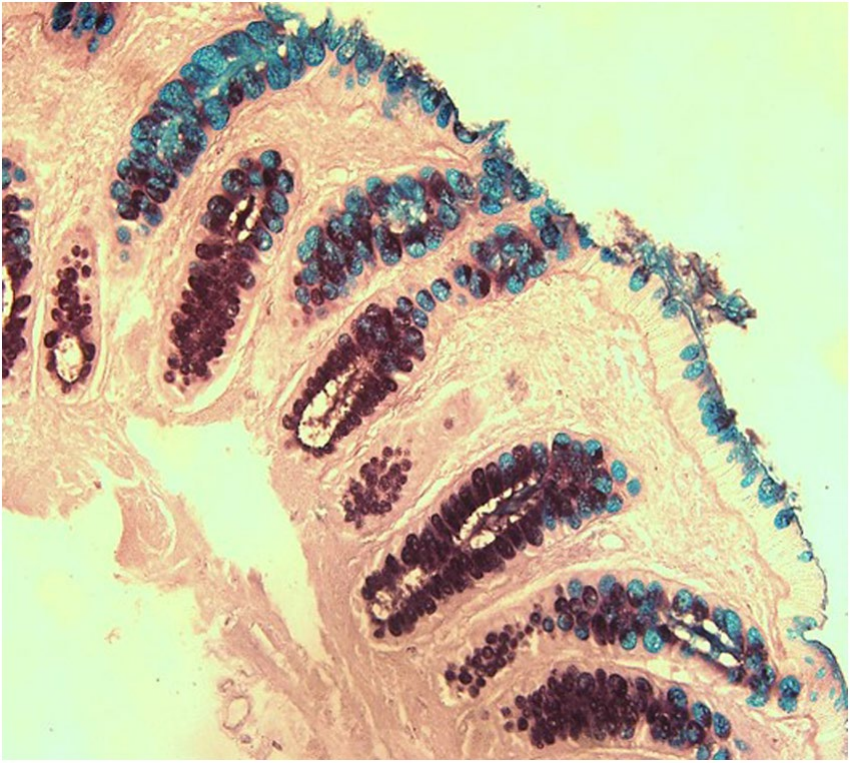
Section of healthy mucosal biopsy stained by HID/AB at magnification 20X. Sulphated mucins are mahogany in colour and sialyated mucins are turquoise blue. The resulting hue, saturation and brightness are used by ImageJ software for colour thresholding^23^.

Formalin fixed, paraffin embedded mucosal biopsy specimens for each mucin sample were stained using Haematoxylin and eosin stain (H&E) and High Iron Diamine-Alcian Blue (HID-AB) staining to quantify degree of inflammation and percentage sulphation respectively as previously described^15,23^ (Fig. 1). For each specimen, the quantity of sulphated mucin was determined and results expressed as the percentage relative to the total mucin content for a given specimen. For histological analysis, UC specimens were scored as mild, moderate or severe inflammation, according to the system described by Geboes *et al*.^24^.

### Construction of plasmid DNA standards

A series of plasmid DNA standards was generated to enable calculation of *A. muciniphila* copy number in each sample. In brief, freeze dried cultures of *A. muciniphila* reference strain ATCC^®^BAA-835 (American Type Culture Collection, Manassas, VA, U.S.A.) were cultured according to manufacturer’s instructions in Brain Heart Infusion (BHI) medium (Sigma Aldrich^®^, Dublin, Ireland). Cultures were placed in a shaking incubator at 200 rpm at 37 °C for 16 hours under anaerobic conditions achieved by the use of AnaeroGen™ anaerobic gas packs (Oxoid, Basingstoke, UK). DNA was extracted using a series of four heat freeze cycles at −80 °C and 100 °C. Conventional PCR targeting the 16S rRNA gene of *A. muciniphila* was performed using oligonucleotide primers targeting *A. muciniphila* (forward primer 5′- CAGCACGTGAAGGTGGGGAC – 3′ reverse primer 5′- CCTTGCGGTTGGCTTCAGAT-3′)^10^. All PCR reactions contained 1X My Taq™ Red Mix (Bioline, London, UK), forward primer and reverse primer at a final concentration of 200 nM. The 327 bp amplicon generated and cloned into a TOPO vector using the TOPO TA cloning system. DNA from the recombinant plasmid mini-preps was purified using the QIAprep Spin MiniPrep kit (Qiagen). The total weight per recombinant plasmid was calculated and this was used to generate a series of DNA standards of known copy number of the target sequence.

### Quantification of *A. muciniphila* in mucus brushings

For each clinical sample the total copy number of bacteria per mg of mucus had previously been determined by quantitative RT-PCR. Conventional PCR analysis targeted the bacterial 16S rRNA gene (forward primer 5′-TCCTACGGGAGGCAGCAGT-3′, reverse primer 5′-GGACTACCAGGGATCT AATCCTGTT-3′) (Eurofins MWG)^25^.

RT PCR using an assay specific for the 16S rRNA gene of *A. muciniphila* was performed using primers detailed above. All reactions were carried out on an Applied Biosystems^®^ 7900HT Fast Real-Time PCR machine (Applied Biosystems^®^ Foster City, CA, USA.). Each reaction was performed in duplicate and carried out in an optical grade 384-well plate at a final volume of 20 µl. Each reaction consisted of 1X Syber^®^Green PCR Master Mix (Applied Biosystems), forward and reverse primers at final concentration of 200 nM and 4 µl of template Standard cycling conditions and melt curve analysis were employed, plus an additional annealing stage at 79 °C for 10 sec.

### Data analysis

Data analysis for PCR assays performed on the Applied Biosystems platform was performed using SDS 2.4 software (Applied Biosystems^®^). Target copy number in each sample was determined based on the fold change (2^−∆Ct^) relative to the 10^7^ DNA standard. Copy numbers were normalised for dilution volume, elution volume, DNA concentration and sample weight. Normalised data were exported to SPSS statistics, version 20.0 (SPSS statistics, IBM^®^, London, U.K.) for statistical analysis. Data were tested for normality of distribution, and statistical comparisons were performed based on Mann-Whitney U test, Spearman-Rho and Kruskal-Wallis comparisons.

## Results

### Total bacterial abundance in the mucus gel layer

**Figure 2:**
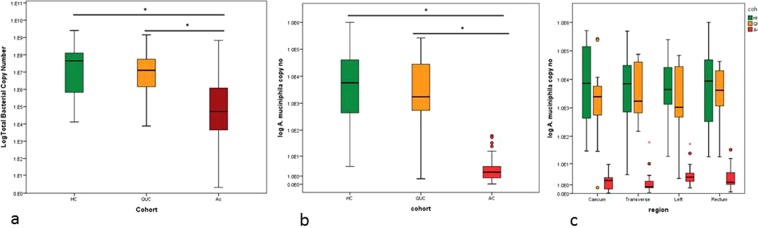
(**a**) Boxplots representing the total bacterial copy number in each cohort. (**b**) Boxplots representing Inter-cohort comparisons of *A. muciniphila* abundance. Patients with acute UC are represented by red, quiescent UC in orange and healthy controls in green. *represents a significant difference (p < 0.05) (**c**) Comparison of *A. muciniphila* copy number across the longitudinal axis of the colon in the three patient cohorts.

Data pertaining to the total bacterial counts in the mucus brush sample were generated using RT-PCR and primers targeting pan-bacterial 16S rRNA gene^20^. Median copy numbers of total bacterial 16S rRNA expressed per mg of mucus are given in Table 1. Subsequent inter-cohort statistical analysis was performed based on the Mann Whitney U test, revealing a reduction in total bacterial copy numbers in AC compared to HC (Fig. 2a, Table 1.) A significant reduction was also observed in AC compared to QUC. No difference was observed between the HC and QUC cohorts. Loco-regional analysis of four colonic areas; caecum, left colon, transverse colon and rectum revealed the same trend (Table 2). These data were used for normalisation of RT-PCR data for *A. muciniphila*, in order to determine the proportional abundance of this microbe in mucus brush samples.

**Table 1:** Median total bacterial copy number/mg of mucus for each patient cohort. p-values of statistical comparisons of total bacterial counts between patient cohorts.

Cohort	n	Median Copy Number	IQR	Cohort Comparison	p-value
HC	20	4.25E + 7	1.26E + 8	**HC vs QUC**	0.176
QUC	13	1.20E + 7	5.68E + 7	**HC vs AC**	**0.000**
AC	20	3.97E + 4	9.99E + 5	**QUC vs AC**	**0.000**

**Table 2:** Total bacterial and *A. muciniphila* copy numbers compared on a Loco-regional basis across the longitudinal axis of the colon.

	n	HC	IQR	n	QUC	IQR	n	AC	IQR	Inter-cohort	Comparisons	QUC-AC
		Median			Median			Median		HC-QUC	HC-AC	
Total Bacterial Copy number												
Caecum	20	5.07E + 7	1.17E + 8	13	1.54E + 7	2.65E + 7	19	1.47E + 5	1.07E + 6	0.065	0.000	0.002
Transverse	19	4.24E + 6	1.94E + 8	13	7.45E + 6	9.61E + 7	19	9.22E + 4	5.40E + 6	0.001	0.001	0.011
Left	19	6.20E + 7	2.12E + 8	13	1.53E + 7	4.21E + 8	19	2.11E + 5	2.68E + 6	0.935	0.001	0.002
Rectum	20	1.74E + 7	6.39E + 7	13	1.27E + 7	6.44E + 6	18	1.24E + 4	3.41E + 5	0.845	0.000	0.000
*A. muciniphila* Copy number												
	**n**	**HC**	**IQR**	**n**	**QUC**	**IQR**	**n**	**AC**	**IQR**	**HC-QUC**	**HC-AC**	**QUC-AC**
		**Median**			**Median**			**Median**				
Caecum	20	8.12E + 03	1.94E + 05	11	2.46E + 03	1.14E + 04	16	1.84E + 00	2.35E + 00	0.364	0.100	0.000
Transverse	18	7.74E + 03	3.87E + 04	11	1.72E + 03	4.70E + 04	13	7.15E-01	2.08E + 00	0.620	0.000	0.000
Left	16	5.44E + 03	3.66E + 04	12	2.40E + 03	3.25E + 04	18	2.74E + 00	3.02E + 00	0.516	0.000	0.000
Rectum	20	2.99E + 03	8.29E + 04	12	2.82E + 03	2.03E + 04	17	1.46E + 00	3.79E + 00	0.640	0.000	0.000
Normalised *A. muciniphila* Copy number												
	**n**	**HC**	**IQR**	**n**	**QUC**	**IQR**	**n**	**AC**	**IQR**	**HC-QUC**	**HC-AC**	**QUC-AC**
		**Median**			**Median**			**Median**				
Caecum	20	1.45E-04	1.21E-02	11	1.85E-04	9.51E-03	16	3.24E-06	1.40E-04	0.741	0.010	0.103
Transverse	18	5.18E-04	7.85E-03	11	2.58E-04	5.45E-04	13	1.29E-06	1.61E-04	0.393	0.028	0.077
Left	16	9.38E-05	8.45E-03	12	8.20E-05	1.67E-03	18	2.08E-05	9.14E-04	0.486	0.190	0.703
Rectum	20	8.12E-04	7.51E-03	12	1.25E-04	2.18E-03	17	2.82E-95	1.72E-03	0.259	0.072	0.425

Comparison of *A. muciniphila* copy number on a Loco-regional basis before and after normalisation against total bacterial copy number.

### Analysis of paired mucosal biopsy samples for the percentage sulphated mucin and inflammatory cell infiltrate

Median values for the percentage sulphated mucin present in each of the three patient cohorts are outlined in Table 3. The proportion of sulphated mucin was reduced in AC compared to HC (p < 0.005) and patients with QUC (p < 0.005).

**Table 3:** Median percentage sulphomucin and inflammatory scores in each of the three patient cohorts.

Cohort		None (n)	Mild/moderate(n)	Severe (n)	Median Sulphomucin (%)	IQR (%)
HC	**Caecum**	15	0	0	91.31	14.9
	**Transverse**	16	0	0		
	**Left**	9	0	0		
	**Rectum**	18	0	0		
QUC	**Caecum**	2	2	0	80.39	29.39
	**Transverse**	0	5	2		
	**Left**	0	2	2		
	**Rectum**	0	4	1		
AC	**Caecum**	1	3	7	47.57	23.49
	**Transverse**	1	4	7		
	**Left**	0	5	9		
	**Rectum**	0	4	8		

Inflammatory scores were grouped into three categories according to their histological scores: no inflammation, mild to moderate inflammation and severe inflammation according to a modified version of the scoring system by Geobes *et al*. A score of 0–1 was deemed none/mild inflammation, a score of 2–3.3 moderate inflammation and 4–4.5 severe inflammation (Table 3).

### Analysis of *A. muciniphila* in the colonic mucus gel layer

Relative quantitation was performed using RT-PCR to determine the copy numbers of the mucolytic species *A. muciniphila* in AC and QUC compared to HC. *A. muciniphila* was detected in all individuals in the AC and HC cohorts and in 13 out of the 14 individuals in the QUC cohort. No difference in *A. muciniphila* raw copy number was observed between the four colonic regions sampled (Kruskall-Wallis p = 0.079).

Statistical comparisons between cohorts were performed using the non-parametric Mann-Whitney U test. *A. muciniphila* was significantly less abundant in the AC patient cohort than in the HC or QUC patient cohort (Fig. 2b, Table 4). No difference was observed between the HC cohort and the QUC patient cohort. Data were normalised against total bacterial copy number. After normalisation, the same trend was observed (Table 4).

**Table 4:** Median copy number/mg and relative abundance of *A. muciniphila* after normalisation for pan bacterial copy number in each patient cohort.

Cohort	n	Median Copy No.	IQR	Cohort Comparison	p value
HC	20	5.73E + 03	4.56E + 04	**HC-QUC**	0.190
QUC	14	1.74E + 03	2.92E + 04	**HC-AC**	**0.000**
AC	20	1.80E + 00	2.89E + 00	**QUC - AC**	**0.000**
**Cohort**	**n**	**Median Normalised Copy No**.	**IQR**	**Cohort Comparison**	**p value**
HC	20	2.58E-03	6.69E-04	**HC-QUC**	0.175
QUC	14	1.86E-04	9.77E-04	**HC-AC**	**0.000**
AC	20	2.04E-05	2.17E-04	**QUC - AC**	**0.020**

p values for inter-cohort comparisons. Significant values are highlighted in bold text.

The reduction in abundance of *A. muciniphila* in the AC cohort compared to HC and QUC was observed in all four areas of the colon, caecum, transverse colon, left colon and rectum (Fig. 2c, Table 2).

After normalisation of data against total bacterial copy number, a significant reduction in *A. muciniphila* was noted in the AC cohort compared to HC in the caecum and transverse colon only (Table 2).

### Correlations between A. muciniphila abundance and inflammatory cell infiltrates and percentage of sulphated mucin

To determine whether the reduction in abundance of *A. muciniphila* in UC was associated with inflammation, correlations with inflammatory scores were performed. These were grouped into three categories according to their histological scores: no inflammation, mild to moderate inflammation and severe inflammation.

Lower abundances of *A. muciniphila* correlated strongly with higher inflammatory scores, as determined by the Spearman Rho correlation (ρ = −0.639 (p < 0.005)). After normalisation of the data against total bacterial copy number a weaker negative correlation was observed (ρ = 0.294 (p = 0.001)).

To determine whether the altered abundance of *A. muciniphila* in the inflamed colon was associated with changes in the proportion of sulphated mucin present, correlations between *A. muciniphila* copy number and the percentage sulphated mucin were performed.

**Figure 3:**
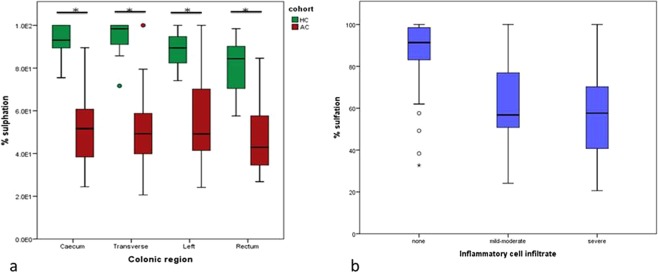
(**a**) Boxplots representing the reduction in the proportion of sulphated mucin present in AC compared to HC across four colonic regions. * indicates a p value of < 0.005 as determined by the Mann Whitney U test. (**b**) Boxplots representing the proportion of sulphomucin present in mucosal samples based on degree of inflammation.

The proportion of sulphated mucin was reduced in the AC cohort compared to healthy controls (p < 0.005) (Fig. 3a). This significant reduction in the acute UC cohort was observed across all four colonic regions examined (Fig. 3a). The distribution of sulfomucin was uniform along the longitudinal axis of the colon (Kruskal Wallis p = 0.174).

The effect of inflammation on degree of mucin sulphation was assessed. Non-inflamed mucosa was associated with the highest percentage of sulphated mucin (Table 5). A significant reduction in the percentage sulphated mucin was observed in mucosa with moderate or severe inflammation compared to no inflammation (p < 0.005) (Fig. 3b, Table 5). No difference was observed in the sulphomucin content between moderate and severely inflamed mucosa (p = 1.00).

**Table 5:** Median percentage sulphomucin in inflamed and non-inflamed mucosa and Spearman Rho correlations with *A. muciniphila* abundance.

Inflammatory cell infiltrate	Median % sulphomucin	IQR
None	9.14E + 2	1.55E + 2
Mild-Moderate	5.68E + 2	2.80E + 2
Severe	5.76E + 2	3.33E + 2
Correlation with *A. muciniphila* abundance	**Correlation coefficient (ρ)**	**P value**
	0.546	**<0.005**

### Correlations between mucin sulphation and A. muciniphila abundance

**Figure 4:**
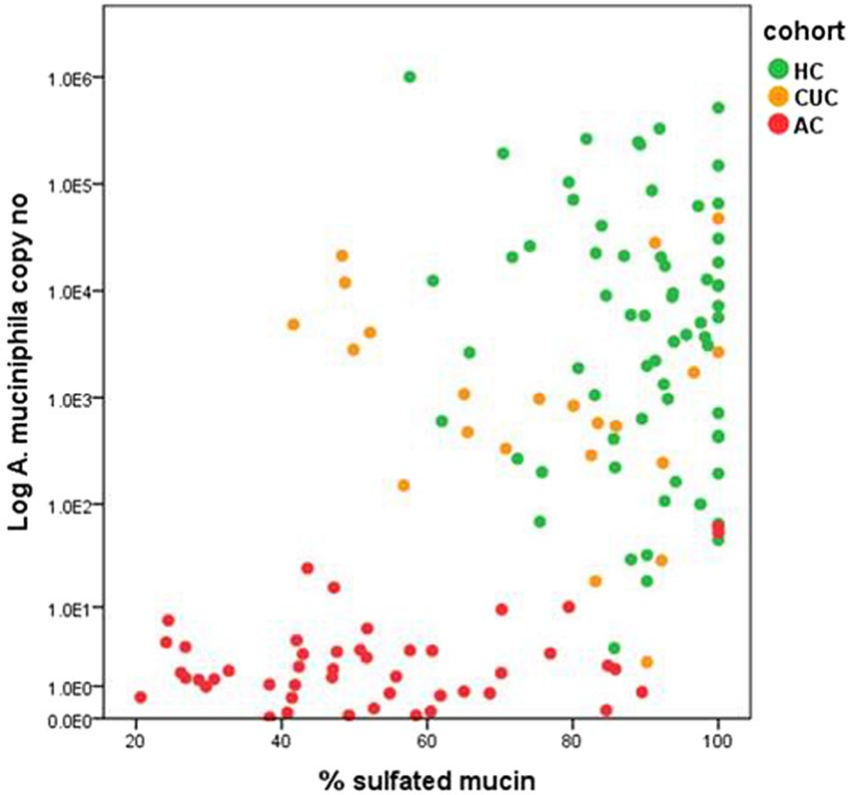
Scatterplot depicting the relationship between *A. muciniphila* abundance and percentage sulphated mucin present in samples. The acute UC cohort is represented by red, quiescent UC by orange and healthy controls in green

A positive association was found between the abundance of *A. muciniphila* and a higher percentage of sulphated mucin (ρ 0.546, p = 0.000) (Fig. 4, Table 6). This association was lost after normalisation of the data (ρ 0.164, p = 0.058).

### Predictors of *A. muciniphila* abundance in health and ulcerative colitis

A summary of patient characteristics is included in supplementary data. Details pertaining to patient demographics are outlined in Supplementary Table 2. To determine whether patient demographic factors influenced *A. muciniphila* abundance, multiple linear regression analysis was performed on all three patient cohorts (Supplementary Table 3). Data were log^10^ transformed prior to analysis to ensure the assumptions of linearity, homoscedasticity and normality were met. Variables assessed included age, gender, smoking status (current, previous, non-smoker) and appendectomy (yes, no). None of the variables examined were predictive of *A. muciniphila* abundance (F = 0.767, p = 0.558). Regression coefficients and p values are outlined in Supplementary data.

A separate analysis was performed on all patients with UC, combining the AC and QUC cohorts, to determine whether drug treatment or Mayo score predicted *A. muciniphila* abundance. Using Spearman Rho correlation, a negative correlation was noted between Mayo score and *A. muciniphila* abundance (ρ = −0.706, p < 0.005).

Multiple linear regression analysis was then performed to predict *A. muciniphila* abundance based on drug treatments (aminosalicylates, biologics, steroids and antibiotics) and Mayo score (F = 7.487, p = 0.001). Mayo score predicted lower *A. muciniphila* abundance (Supplementary Table 3). Abundance did not vary with treatment modality (Supplementary Table 3).

## Discussion

A high rate of colonisation with *A. muciniphila* was observed in all three patient cohorts, indicating that this species is a common commensal inhabiting the MGL of the human colon. *A. muciniphila* has been widely detected in faecal samples and mucosal biopsies^1,3,26^, however to the authors’ knowledge, this is the first study to quantitatively analyse this species in mucus brushings of the colonic mucus. No trend in *A. muciniphila* abundance was evident along the longitudinal axis of the colon, in keeping with current literature supporting spatial homogeneity of the commensal microbiota extending from caecum to rectum^19^.

These data indicate a reduction in the abundance of *A. muciniphila* within the MGL in AC compared to health and are in keeping with the published literature^10^. In QUC, the abundance returns towards levels observed in the healthy colon, suggesting that the reduced abundance is related to the inflammatory process, rather than being a feature of the microbial signature of individuals suffering from UC. Normalisation of data against total bacterial copy number reduced potential reporting errors by minimising the effect of between-sample variation and taking the efficiency of the quantification procedure into account. These data are more representative of the actual burden of the target in the MGL. However, normalisation negated the significant difference in abundance observed between health and acute UC when analysis was based on raw copy numbers. This is likely due to the fact that *A. muciniphila* accounts for a small proportion (thought to be 1–3%) of the overall bacterial load in the colon^1,3^. These estimates from the literature were based on analysis of faecal samples. The normalised data presented here suggest that the overall abundance of this species in the MGL is lower than this.

The present study uses protected specimen brushings (PSB) of the colonic mucosa as the sampling method, reducing potential contamination from luminal contents or mucosal associated bacteria during insertion and withdrawal^20^, therefore the findings are likely to represent the true burden of these species within the MGL. It is possible that the use of bowel preparation may have resulted in a loss of some loosely adherent microbes in the patients undergoing colonscopy (healthy controls and quiescent cohorts), however, this would not account for the low colonisation rates in patients with acute UC, as this cohort did not receive bowel preparation prior to surgery. Despite the fact that the true burden of *A. muciniphila* in the colon is low, this microbe has the ability to affect host gene expression,^6,27^ therefore alterations in the abundance of this species in disease states may nonetheless have implications for the host.

Johansson *et al*., demonstrated that in UC, bacteria can penetrate the normally sterile inner layer of the MGL^28^, a fact which should also be considered when interpreting these data and may account for the observed reduction in *A. muciniphila* in the AC cohort.

It should be noted that all patients included in this study were fasting for a minimum of 24 hours prior to biopsy collection. *A. muciniphila* does not rely on dietary substances for substrate and consequently is conferred with a competitive advantage during periods of fasting^29,30^. The abundances reported here, therefore, may not be truly representative of the normal healthy colon. This potential confounding factor would be difficult to eliminate, as adequate bowel cleansing is a prerequisite for colonoscopy. Furthermore, as all three groups included in this study were fasting, comparisons of the relative abundances between cohorts should not be affected.

The second aim of this study was to assess if a correlation exists between *A. muciniphila* and the degree of mucin sulphation present in mucosal biopsies. One possible explanation for reduced abundance AC is a lack of substrate. *A. muciniphila* has the capacity to produce sulfatases^17^ and may use sulphated mucin as their substrate. Analysis of the sulphomucin profiles in the three cohorts indicated that the inflamed mucosa in acute UC was associated with a significantly lower percentage of sulphomucin. Lower percentage of sulphomucin was associated with reduced *A. muciniphila* abundance, and multiple linear regression analysis revealed that percentage sulphation significantly predicted *A. muciniphila* abundance, indicating a possible link between sulphomucin content and *A. muciniphila* burden. These data lend support to the hypothesis that a lack of sulphomucin substrate may contribute to the reduced abundance of this microbe in the inflamed colon. Of note, a correlation between the species *Desulfovibrio*, a species also capable of degrading sulphated mucin, and reduced sulphated mucin in the colitic colon has previously been demonstrated lending support to the hypothesis that alterations in mucin biochemistry may contribute of changes in the microbiota observed in UC^15^.

It is likely that the lack of sulphated mucin in AC is not the sole contributor to alterations in *A. muciniphila* abundance. Other biochemical changes in mucin composition have been reported in UC, including reduced levels of MUC2 and alterations in glycosylation^16,31^, which may also influence microbial survival and proliferation.

The results also show that higher inflammatory scores were associated with reduced abundances of *A. muciniphila*. In keeping with this, the MAYO score was the only clinical parameter that was predictive of *A. muciniphila* abundance. It is conceivable that bacterial growth inhibition resulting from the inflammatory process itself may account for the reduced abundance of *A. muciniphila* in UC. The production of inflammatory mediators and associated changes in the micro-environment may render the MGL a less hospitable niche for this commensal. UC is also associated with increased colonisation with opportunistic pathogens, such as members of the Enterobacteriaceae family^32^, which could potentially lead to competitive exclusion of *A. muciniphila. In vitro* studies have demonstrated growth inhibition of *A. muciniphila* in co-culture compared to pure culture, lending support to this hypothesis^10^. In contrast to these findings however, one study in the literature reported exacerbation of Salmonella typhimurium induced inflammation in the presence of *A. muciniphila*^33^.

In addition to mucin degradation^10,17^, *A. muciniphila* may have other functions which are beneficial to the host^7,34–36^. Mechanisms that have been proposed include: production of essential SCFAs such as propionate and acetate as a result of mucin degradation^17^, immunomodulation of the adaptive immune system^6^, protective barrier functions^7,37^ and anti-inflammatory properties^38^. The role of *A. muciniphila* in modulating metabolic pathways has been well described in obesity, diabetes and other cardiometabolic disorders^4,27,35,39–41^, conditions which, like UC, are associated with an altered microbiota, inflammation and altered gut barrier function. Derangements in the abundance of this microbe may have important metabolic implications in the colon and warrant further investigation in the setting of UC.

*A. muciniphila* may be involved in a positive feedback loop, whereby through mucin degradation it stimulates mucin production and renewal of the MGL^34^. While this has not been described specifically in the context of UC, evidence suggests that the species has the ability to ameliorate age related depletion of colonic mucus^42^ suggesting that this loop may exist at times of stress or disease states. If this hypothesis holds true, then reduced activity of this species may represent a primary pathogenic mechanism in UC. In contrast to the described beneficial actions, one study in murine models demonstrated a link between *A. muciniphila* colonisation and development of colitis. However this may have been attributable to loss of host immune tolerance to commensal microbiota in disease states, rather than a true pathogenic mechanism of *A. muciniphila*^43^.

Overall, this study lends support to the hypothesis that *A. muciniphila* is a symbiont member of the human colonic microbiota and confirms an inverse relationship between its abundance in the MGL and active inflammation. The observed reduction in abundance may be the result of an altered micro-environment in the inflamed colon itself, however these data lend support to the hypothesis that it is a consequence of reduced availability of sulphated mucin substrate.

## Supplementary information

Supplementary Information

## References

[1] Derrien M, Collado MC, Ben-Amor K, Salminen S, de Vos WM (2008). The Mucin degrader Akkermansia muciniphila is an abundant resident of the human intestinal tract. Applied and environmental microbiology.

[2] van Passel MW (2011). The genome of Akkermansia muciniphila, a dedicated intestinal mucin degrader, and its use in exploring intestinal metagenomes. PloS one.

[3] Collado MC, Derrien M, Isolauri E, de Vos WM, Salminen S (2007). Intestinal integrity and Akkermansia muciniphila, a mucin-degrading member of the intestinal microbiota present in infants, adults, and the elderly. Applied and environmental microbiology.

[4] Clarke SF (2014). Exercise and associated dietary extremes impact on gut microbial diversity. Gut.

[5] Guo X (2016). Different subtype strains of Akkermansia muciniphila abundantly colonize in southern China. Journal of applied microbiology.

[6] Derrien M (2011). Modulation of Mucosal Immune Response, Tolerance, and Proliferation in Mice Colonized by the Mucin-Degrader Akkermansia muciniphila. Frontiers in microbiology.

[7] Reunanen Justus, Kainulainen Veera, Huuskonen Laura, Ottman Noora, Belzer Clara, Huhtinen Heikki, de Vos Willem M., Satokari Reetta (2015). Akkermansia muciniphila Adheres to Enterocytes and Strengthens the Integrity of the Epithelial Cell Layer. Applied and Environmental Microbiology.

[8] James SL (2015). Abnormal fibre usage in UC in remission. Gut.

[9] Derrien Muriel, Belzer Clara, de Vos Willem M. (2017). Akkermansia muciniphila and its role in regulating host functions. Microbial Pathogenesis.

[10] Png CW (2010). Mucolytic bacteria with increased prevalence in IBD mucosa augment *in vitro* utilization of mucin by other bacteria. The American journal of gastroenterology.

[11] Papa E (2012). Non-Invasive Mapping of the Gastrointestinal Microbiota Identifies Children with Inflammatory Bowel Disease. PloS one.

[12] Vigsnaes LK, Brynskov J, Steenholdt C, Wilcks A, Licht TR (2012). Gram-negative bacteria account for main differences between faecal microbiota from patients with ulcerative colitis and healthy controls. Beneficial microbes.

[13] Johansson ME, Larsson JM, Hansson GC (2011). The two mucus layers of colon are organized by the MUC2 mucin, whereas the outer layer is a legislator of host-microbial interactions. Proceedings of the National Academy of Sciences of the United States of America.

[14] Raouf AH (1992). Sulphation of colonic and rectal mucin in inflammatory bowel disease: reduced sulphation of rectal mucus in ulcerative colitis. Clinical science (London, England: 1979).

[15] Lennon G (2014). Correlations between colonic crypt mucin chemotype, inflammatory grade and Desulfovibrio species in ulcerative colitis. Colorectal disease: the official journal of the Association of Coloproctology of Great Britain and Ireland.

[16] Larsson JM (2011). Altered O-glycosylation profile of MUC2 mucin occurs in active ulcerative colitis and is associated with increased inflammation. Inflammatory bowel diseases.

[17] Derrien M, Vaughan EE, Plugge CM, de Vos WM (2004). Akkermansia muciniphila gen. nov., sp. nov., a human intestinal mucin-degrading bacterium. International journal of systematic and evolutionary microbiology.

[18] Zoetendal EG (2002). Mucosa-associated bacteria in the human gastrointestinal tract are uniformly distributed along the colon and differ from the community recovered from feces. Applied and environmental microbiology.

[19] Lavelle A, Lennon G, O'Sullivan O, Docherty N, Balfe A, Maguire A, Mulcahy H E, Doherty G, O'Donoghue D, Hyland J, Ross R P, Coffey J C, Sheahan K, Cotter P D, Shanahan F, Winter D C, O'Connell P R (2015). Spatial variation of the colonic microbiota in patients with ulcerative colitis and control volunteers. Gut.

[20] Lavelle A (2013). Depth-dependent differences in community structure of the human colonic microbiota in health. PloS one.

[21] Huse SM (2014). Comparison of brush and biopsy sampling methods of the ileal pouch for assessment of mucosa-associated microbiota of human subjects. Microbiome.

[22] Schroeder KW, Tremaine WJ, Ilstrup DM (1987). Coated oral 5-aminosalicylic acid therapy for mildly to moderately active ulcerative colitis. A randomized study. The New England journal of medicine.

[23] Balfe, A. Profiling the Transcriptional Signature of Colonic Mucosa and Epithelial Cells of Patients with Ulcerative Colitis and a Study of the Translated Effects of these Signatures (University College Dublin, 2015).

[24] Geboes K (2000). A reproducible grading scale for histological assessment of inflammation in ulcerative colitis. Gut.

[25] Nadkarni MA, Martin FE, Jacques NA, Hunter N (2002). Determination of bacterial load by real-time PCR using a broad-range (universal) probe and primers set. Microbiology (Reading, England).

[26] Lopez-Siles M (2018). Alterations in the Abundance and Co-occurrence of Akkermansia muciniphila and Faecalibacterium prausnitzii in the Colonic Mucosa of Inflammatory Bowel Disease Subjects. Frontiers in cellular and infection microbiology.

[27] Lukovac, S. *et al*. Differential modulation by Akkermansia muciniphila and Faecalibacterium prausnitzii of host peripheral lipid metabolism and histone acetylation in mouse gut organoids. **5**, 10.1128/mBio.01438-14 (2014). 10.1128/mBio.01438-14 PMC4145684 25118238

[28] Johansson ME (2014). Bacteria penetrate the normally impenetrable inner colon mucus layer in both murine colitis models and patients with ulcerative colitis. Gut.

[29] Sonoyama K (2009). Response of gut microbiota to fasting and hibernation in Syrian hamsters. Applied and environmental microbiology.

[30] Remely M (2015). Increased gut microbiota diversity and abundance of Faecalibacterium prausnitzii and Akkermansia after fasting: a pilot study. Wiener klinische Wochenschrift.

[31] Theodoratou Evropi, Campbell Harry, Ventham Nicholas T., Kolarich Daniel, Pučić-Baković Maja, Zoldoš Vlatka, Fernandes Daryl, Pemberton Iain K., Rudan Igor, Kennedy Nicholas A., Wuhrer Manfred, Nimmo Elaine, Annese Vito, McGovern Dermot P. B., Satsangi Jack, Lauc Gordan (2014). The role of glycosylation in IBD. Nature Reviews Gastroenterology & Hepatology.

[32] Willing BP (2010). A pyrosequencing study in twins shows that gastrointestinal microbial profiles vary with inflammatory bowel disease phenotypes. Gastroenterology.

[33] Ganesh BP, Klopfleisch R, Loh G, Blaut M (2013). Commensal Akkermansia muciniphila exacerbates gut inflammation in Salmonella Typhimurium-infected gnotobiotic mice. PloS one.

[34] Belzer C, de Vos WM (2012). Microbes inside–from diversity to function: the case of Akkermansia. The ISME journal.

[35] Shin NR (2014). An increase in the Akkermansia spp. population induced by metformin treatment improves glucose homeostasis in diet-induced obese mice. Gut.

[36] Ottman N, Geerlings SY, Aalvink S, de Vos WM, Belzer C (2017). Action and function of Akkermansia muciniphila in microbiome ecology, health and disease. Best practice & research. Clinical gastroenterology.

[37] Ottman N (2017). Pili-like proteins of Akkermansia muciniphila modulate host immune responses and gut barrier function. PloS one.

[38] Kang CS (2013). Extracellular Vesicles Derived from Gut Microbiota, Especially Akkermansia muciniphila, Protect the Progression of Dextran Sulfate Sodium-Induced Colitis. PloS one.

[39] Everard A (2013). Cross-talk between Akkermansia muciniphila and intestinal epithelium controls diet-induced obesity. Proceedings of the National Academy of Sciences of the United States of America.

[40] Dao MC (2016). Akkermansia muciniphila and improved metabolic health during a dietary intervention in obesity: relationship with gut microbiome richness and ecology. Gut.

[41] Plovier Hubert, Everard Amandine, Druart Céline, Depommier Clara, Van Hul Matthias, Geurts Lucie, Chilloux Julien, Ottman Noora, Duparc Thibaut, Lichtenstein Laeticia, Myridakis Antonis, Delzenne Nathalie M, Klievink Judith, Bhattacharjee Arnab, van der Ark Kees C H, Aalvink Steven, Martinez Laurent O, Dumas Marc-Emmanuel, Maiter Dominique, Loumaye Audrey, Hermans Michel P, Thissen Jean-Paul, Belzer Clara, de Vos Willem M, Cani Patrice D (2016). A purified membrane protein from Akkermansia muciniphila or the pasteurized bacterium improves metabolism in obese and diabetic mice. Nature Medicine.

[42] van der Lugt B (2019). Akkermansia muciniphila ameliorates the age-related decline in colonic mucus thickness and attenuates immune activation in accelerated aging Ercc1 (-/Delta7) mice. Immunity & ageing: I & A.

[43] Seregin SS (2017). NLRP6 Protects Il10(-/-) Mice from Colitis by Limiting Colonization of Akkermansia muciniphila. Cell reports.

