# Supplementary material for: The abundance of Akkermansia muciniphila and its relationship with sulphated colonic mucins in health and ulcerative colitis
Source: Sci Rep. 2019 Oct 30;9:15683. doi: 10.1038/s41598-019-51878-3 (PMC6821857; doi:10.1038/s41598-019-51878-3)
Supplement: Supplementary file 1 — Supplementary Information [file 41598_2019_51878_MOESM1_ESM.pdf]

# The abundance of *Akkermansia muciniphila* and its relationship with sulphated colonic mucins in health and ulcerative colitis

Helen Earley[1,2,*+], Grainne Lennon[1,2+], Áine Balfe[1,2], J. Calvin Coffey [3], Desmond C Winter[2], P. Ronan O'Connell[1,2].

[1.] School of Medicine, University College Dublin, Belfield, Dublin 4, Ireland
[2.] Centre for Colorectal Disease, St Vincent's University Hospital, Dublin 4, Ireland
[3.] Graduate Entry Medical School, Limerick, Ireland
*Corresponding Author helen.earley@ucdconnect.ie
[+]These authors contributed equally to this work

| Clinical Finding | Score |
| --- | --- |
| **No. of Bowel Motions/day** | |
| Normal for the patient | 0 |
| 1-2 more than normal | 1 |
| 3-4 more than normal | 2 |
| 5 or greater more than normal | 3 |
| **Presence of Blood** | |
| No blood | 0 |
| Streaks less than half the time | 1 |
| Obvious blood most of the time | 2 |
| Frank blood a | 3 |
| **Endoscopic Findings** | |
| Normal or inactive disease | 0 |
| Mild disease | 1 |
| Moderate disease | 2 |
| Severe disease | 3 |
| **Clinician Global Assessment** | |
| Normal | 0 |
| Mild disease | 1 |
| Moderate disease | 2 |
| Severe disease | 3 |

**Table S1.** Mayo Scoring System for Assessment of Ulcerative Colitis Activity

## Patient Demographics

Fifty-four individuals were included in the study in total, comprising 20 patients with AC, 14 with QUC and 20 HC. Patients in the AC group had undergone colectomy for disease refractory to medical management. No patients in this group had presented with an acute surgical emergency such as toxic megacolon. Of the patients in the quiescent group, 5 underwent colonoscopy to monitor disease activity, the remaining 9 patients were undergoing screening colonoscopy procedures, eight of whom had disease duration of greater than ten years. As a result, the mean time since diagnosis was longer in the quiescent group than in the acute colitis group. In addition, the mean age in the QUC cohort (49yrs) was also older than in the AC cohort (36yrs). The mean age in the HC cohort was 48.2yrs which closely resembled that of the quiescent cohort. The mean Mayo score of AC patients was 10.3, whereas the QUC cohort had a mean of 0.25. Information with regard to smoking status, medication and previous appendectomy was available. A summary of patient demographics is outlined in Supplementary Table 2.

| | | HC | QUC | AC |
|---|---|---|---|---|
| **n** | | 20 | 14 | 20 |
| **Age (years)** | | 48.2 (21-77) | 50.07 (29-72) | 37.4 (23-66) |
| **Gender (M/F)** | | 16/4 | 10/4 | 12/8 |
| **Time since diagnosis (years)** | | n/a | 18 (10-33) | 5 (2-14) |
| **Appendectomy** | | 1 | 1 | 3 |
| **Smoking Status** | Current | 0 | 6 | 2 |
| | Former | 4 | | 3 |
| | Never | 9 | 2<br>6 | 9 |
| **Medication use** | Steroid | n/a | 1 | 12 |
| | 5-ASA | n/a | 7 | 8 |
| | Azathioprine | n/a | 4 | 0 |
| | Infliximab | n/a | 0 | 12 |
| **Mean Mayo Score** | | 0 | 0.25 | 10.3 |

**Table S2**. Summary of patient demographics and characteristics of individuals in each cohort

| Variable | *B* | *SE<sub>B</sub>* | β | t value | p |
|---|---|---|---|---|---|
| **Intercept** | 1.956 | 2.712 | | | |
| **Age** | 0.033 | 0.022 | 0.317 | 1.475 | 0.154 |
| **Gender** | -0.067 | 1.000 | -0.014 | -0.067 | 0.947 |
| **Smoking status** | -0.268 | 0.709 | -0.079 | -0.378 | 0.709 |
| **Appendectomy** | 0.251 | 0.875 | 0.058 | 0.287 | 0.777 |
| **Variable** | *B* | $SE_B$ | β | t value | p |
| **Intercept** | 3.985 | 4.233 | -0.844 | 0.941 | 0.361 |
| **MAYO score** | -0.367 | 0.148 | -0.021 | -2.489 | **0.025** |
| **Biologic Tx** | 0.417 | 1.333 | 0.102 | 0.353 | 0.729 |
| **Steroids** | -0.088 | 1.097 | -0.021 | -0.080 | 0.937 |
| **Aminosalicylate** | 0.634 | 0.809 | 0.151 | 0.784 | 0.445 |
| **Antibiotic** | -0.939 | 1.722 | -0.096 | -0.594 | 0.594 |

**Table S3.** Summary of multiple regression analysis and p values predicting *A. muciniphila* abundance. B = unstandardized regression coefficient, $SE_B$ = standard error of the coefficient, β = standardized coefficient
